# Dimethyl[(*E*)-(2-nitromethylidene-1,3-dithiolan-4-yl)methyl]amine

**DOI:** 10.1107/S1600536812021307

**Published:** 2012-05-16

**Authors:** Shuang-Hua Yang, Zhi-Wei Zhai

**Affiliations:** a Department of Environment Engineering and Chemistry, Luoyang Institute of Science and Technology, Luoyang 471023, People’s Republic of China

## Abstract

In the title compound, C_7_H_12_N_2_O_2_S_2_, the conformation of the dithia­cyclo­pentane ring is a half-chair, with a total puckering amplitude *Q*_T_ = 0.473 (5) Å. Inter­molecular C—H⋯N and C—H⋯O inter­actions help to establish the packing.

## Related literature

For the crystal structures of related compounds, see: Xu *et al.* (2005 ▶); Ortega-Jimenez *et al.* (2000 ▶). For the biological activities of heterocyclic compounds, see: Xu *et al.* (2006 ▶); Yu *et al.* (2009 ▶). For puckering amplitude, see: Cremer & Pople (1975 ▶).
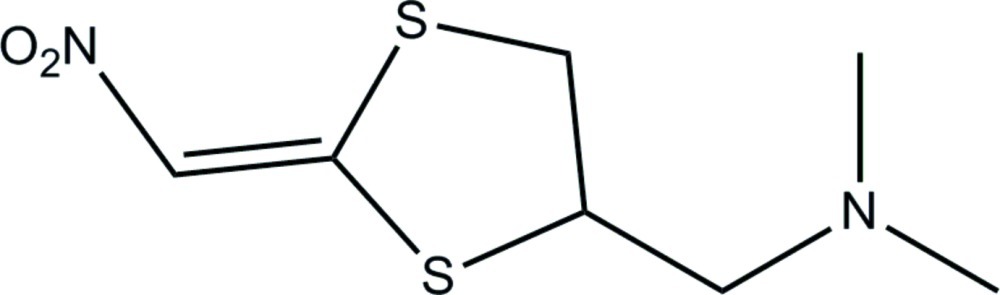


## Experimental

### 

#### Crystal data

C_7_H_12_N_2_O_2_S_2_
*M* *_r_* = 220.31
Orthorhombic, 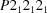

*a* = 5.927 (4) Å
*b* = 11.241 (8) Å
*c* = 14.90 (1) Å
*V* = 992.7 (11) Å^3^
*Z* = 4
Mo *K*α radiation
μ = 0.51 mm^−1^
*T* = 113 K
0.30 × 0.18 × 0.08 mm

#### Data collection

Rigaku Saturn CCD area-detector diffractometer
Absorption correction: multi-scan (*CrystalClear*; Rigaku, 2007 ▶) *T*_min_ = 0.863, *T*_max_ = 0.961
10308 measured reflections
2346 independent reflections
2099 reflections with *I* > 2σ(*I*)
*R*_int_ = 0.051

#### Refinement

*R*[*F* ^2^ > 2σ(*F* ^2^)] = 0.043
*wR*(*F* ^2^) = 0.090
*S* = 1.02
2346 reflections
120 parameters
H-atom parameters constrained
Δρ_max_ = 0.48 e Å^−3^
Δρ_min_ = −0.39 e Å^−3^
Absolute structure: Flack (1983 ▶), 963 Friedel pairs
Flack parameter: −0.02 (10)

### 

Data collection: *CrystalClear* (Rigaku, 2007 ▶); cell refinement: *CrystalClear*; data reduction: *CrystalClear*; program(s) used to solve structure: *SHELXS97* (Sheldrick, 2008 ▶); program(s) used to refine structure: *SHELXL97* (Sheldrick, 2008 ▶); molecular graphics: *SHELXL97*; software used to prepare material for publication: *SHELXTL* (Sheldrick, 2008 ▶).

## Supplementary Material

Crystal structure: contains datablock(s) I, global. DOI: 10.1107/S1600536812021307/hg5213sup1.cif

Structure factors: contains datablock(s) I. DOI: 10.1107/S1600536812021307/hg5213Isup2.hkl

Supplementary material file. DOI: 10.1107/S1600536812021307/hg5213Isup3.cml

Additional supplementary materials:  crystallographic information; 3D view; checkCIF report

## Figures and Tables

**Table 1:** Hydrogen-bond geometry (Å, °)

*D*—H⋯*A*	*D*—H	H⋯*A*	*D*⋯*A*	*D*—H⋯*A*
C1—H1⋯N2^i^	0.95	2.44	3.354 (4)	161
C4—H4⋯O1^ii^	1.00	2.44	3.272 (4)	140

Symmetry codes: (i) 
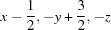
; (ii) 
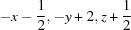
.

**Fig. 1.**
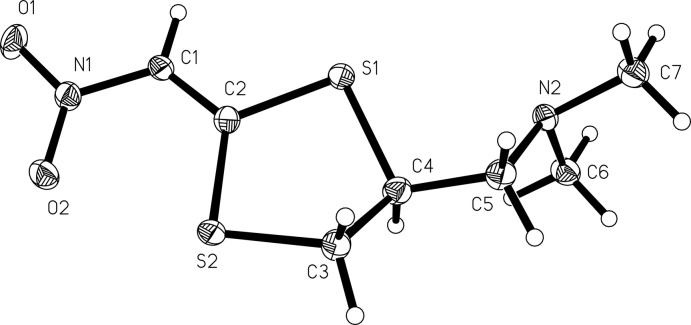
The molecular structure of (I), with atom labels and 40% probability displacement ellipsoids for non-H atoms.

## supplementary crystallographic information

### Comment

Many heterocyclic compounds have been widely used as potent and broad-spectrum fungicides (Xu *et al.*, 2006; Yu *et al.*, 2009). In order to search for new heterocylic compounds with higher biological activities, we synthesized the (*E*)—*N*,*N*-dimethyl-1-(2-(nitromethylene)-1,3-dithiolan-4-yl)methanamine and describe its structure here.

In the title compound, all bond lengths in the molecular are normal and in good agreement with those reported previously (Xu *et al.*, 2005; Ortega-Jimenez *et al.*, 2000). The conformation of the dithiacyclopentane ring (C2—C4/S1/S2) is halfchair, with a total puckering amplitude (Cremer & Pople, 1975) Q_T_ = 0.456 (3) Å and a pseudo-twofold axis running along the direction through C2 and the mid-point of the C3—C4 bond. The intermolecular C—H···N and C—H···O hydrogen bonds stabilize the structure.

### Experimental

A mixture of potassium 2-nitroethene-1,1-bis(thiolate) 10 mmol (2.13 g), 2,3-dichloro-*N*,*N*-dimethylpropan-1-amine (1.56 g, 10 mmol) is refluxed in absolute ethanol (25 ml) for 3 h. The mixture was filtered to provide crude product. The residue was purified by recrystallized from absolute EtOH, yield 1.89 g (86.0%). Single crystals suitable for X-ray measurements were obtained by recrystallization from acetonitrile at room temperature.

### Refinement

H atoms were positioned geometrically and refined using a riding model, with C—H = 0.95 or 1.00 Å and with *U*_iso_(H) = 1.2 times *U*_eq_(C) for methylene H atoms and 1.5*U*_eq_(C) for the methyl H atoms.

### Crystal data

**Table:**

C_7_H_12_N_2_O_2_S_2_	*F*(000) = 464
*M**_r_* = 220.31	*D*_x_ = 1.474 Mg m^−^^3^
Orthorhombic, *P*2_1_2_1_2_1_	Mo *K*α radiation, λ = 0.71073 Å
Hall symbol: P 2ac 2ab	Cell parameters from 3456 reflections
*a* = 5.927 (4) Å	θ = 1.8–27.8°
*b* = 11.241 (8) Å	µ = 0.51 mm^−^^1^
*c* = 14.90 (1) Å	*T* = 113 K
*V* = 992.7 (11) Å^3^	Prism, colorless
*Z* = 4	0.30 × 0.18 × 0.08 mm

### Data collection

**Table:**

Rigaku Saturn CCD area-detector diffractometer	2346 independent reflections
Radiation source: rotating anode	2099 reflections with *I* > 2σ(*I*)
Multilayer monochromator	*R*_int_ = 0.051
Detector resolution: 14.63 pixels mm^-1^	θ_max_ = 27.8°, θ_min_ = 2.3°
ω and φ scans	*h* = −6→7
Absorption correction: multi-scan (*CrystalClear*; Rigaku, 2007)	*k* = −14→14
*T*_min_ = 0.863, *T*_max_ = 0.961	*l* = −19→19
10308 measured reflections	

### Refinement

**Table:**

Refinement on *F*^2^	Secondary atom site location: difference Fourier map
Least-squares matrix: full	Hydrogen site location: inferred from neighbouring sites
*R*[*F*^2^ > 2σ(*F*^2^)] = 0.043	H-atom parameters constrained
*wR*(*F*^2^) = 0.090	*w* = 1/[σ^2^(*F*_o_^2^) + (0.0465*P*)^2^] where *P* = (*F*_o_^2^ + 2*F*_c_^2^)/3
*S* = 1.02	(Δ/σ)_max_ < 0.001
2346 reflections	Δρ_max_ = 0.48 e Å^−^^3^
120 parameters	Δρ_min_ = −0.39 e Å^−^^3^
0 restraints	Absolute structure: Flack (1983), 963 Friedel pairs
Primary atom site location: structure-invariant direct methods	Flack parameter: −0.02 (10)

### Special details

**Table:**

Geometry. All e.s.d.'s (except the e.s.d. in the dihedral angle between two l.s. planes) are estimated using the full covariance matrix. The cell e.s.d.'s are taken into account individually in the estimation of e.s.d.'s in distances, angles and torsion angles; correlations between e.s.d.'s in cell parameters are only used when they are defined by crystal symmetry. An approximate (isotropic) treatment of cell e.s.d.'s is used for estimating e.s.d.'s involving l.s. planes.
Refinement. Refinement of *F*^2^ against ALL reflections. The weighted *R*-factor *wR* and goodness of fit *S* are based on *F*^2^, conventional *R*-factors *R* are based on *F*, with *F* set to zero for negative *F*^2^. The threshold expression of *F*^2^ > σ(*F*^2^) is used only for calculating *R*-factors(gt) *etc*. and is not relevant to the choice of reflections for refinement. *R*-factors based on *F*^2^ are statistically about twice as large as those based on *F*, and *R*- factors based on ALL data will be even larger.

### Fractional atomic coordinates and isotropic or equivalent isotropic displacement parameters (Å^2^)

**Table:**

	*x*	*y*	*z*	*U*_iso_*/*U*_eq_	
S1	0.16147 (11)	0.88829 (5)	0.02451 (4)	0.02114 (16)	
S2	−0.05675 (11)	1.12418 (5)	0.02804 (4)	0.02176 (16)	
O1	−0.4710 (3)	0.96345 (17)	−0.18337 (13)	0.0307 (5)	
O2	−0.3988 (3)	1.10926 (16)	−0.09091 (12)	0.0276 (4)	
N1	−0.3632 (4)	1.00756 (17)	−0.12006 (14)	0.0222 (5)	
N2	0.4568 (4)	0.81812 (18)	0.18117 (14)	0.0196 (5)	
C1	−0.1909 (5)	0.9375 (2)	−0.08113 (16)	0.0203 (5)	
H1	−0.1734	0.8573	−0.0999	0.024*	
C2	−0.0496 (4)	0.9819 (2)	−0.01767 (17)	0.0192 (5)	
C3	0.1989 (5)	1.1078 (2)	0.09357 (18)	0.0256 (6)	
H3A	0.3319	1.1288	0.0566	0.031*	
H3B	0.1945	1.1617	0.1461	0.031*	
C4	0.2158 (4)	0.9797 (2)	0.12456 (17)	0.0225 (6)	
H4	0.0973	0.9636	0.1708	0.027*	
C5	0.4449 (5)	0.9457 (2)	0.16155 (18)	0.0247 (6)	
H5A	0.5630	0.9667	0.1173	0.030*	
H5B	0.4743	0.9914	0.2172	0.030*	
C6	0.3446 (5)	0.7885 (2)	0.26605 (17)	0.0255 (6)	
H6A	0.3518	0.7023	0.2759	0.038*	
H6B	0.1865	0.8137	0.2634	0.038*	
H6C	0.4206	0.8295	0.3156	0.038*	
C7	0.6931 (4)	0.7797 (3)	0.18472 (18)	0.0261 (6)	
H7A	0.7689	0.8189	0.2352	0.039*	
H7B	0.7688	0.8014	0.1286	0.039*	
H7C	0.6995	0.6933	0.1928	0.039*	

### Atomic displacement parameters (Å^2^)

**Table:**

	*U*^11^	*U*^22^	*U*^33^	*U*^12^	*U*^13^	*U*^23^
S1	0.0270 (3)	0.0141 (3)	0.0223 (3)	0.0024 (3)	−0.0050 (3)	−0.0022 (3)
S2	0.0298 (4)	0.0134 (3)	0.0220 (3)	0.0023 (3)	−0.0032 (3)	−0.0015 (3)
O1	0.0375 (12)	0.0268 (10)	0.0279 (10)	−0.0037 (9)	−0.0164 (9)	−0.0017 (8)
O2	0.0313 (11)	0.0185 (9)	0.0330 (10)	0.0037 (8)	−0.0065 (8)	−0.0030 (8)
N1	0.0268 (12)	0.0167 (10)	0.0231 (11)	−0.0006 (9)	−0.0035 (10)	0.0016 (8)
N2	0.0235 (11)	0.0156 (10)	0.0198 (11)	0.0034 (9)	−0.0015 (9)	0.0023 (8)
C1	0.0270 (14)	0.0140 (11)	0.0198 (12)	0.0031 (11)	−0.0008 (11)	0.0006 (9)
C2	0.0219 (13)	0.0180 (11)	0.0177 (12)	−0.0001 (10)	0.0007 (11)	0.0028 (10)
C3	0.0344 (15)	0.0145 (11)	0.0279 (13)	−0.0005 (11)	−0.0100 (12)	−0.0005 (10)
C4	0.0263 (15)	0.0189 (12)	0.0221 (13)	−0.0007 (11)	−0.0016 (11)	−0.0007 (10)
C5	0.0296 (15)	0.0167 (12)	0.0277 (14)	−0.0022 (12)	−0.0055 (13)	−0.0007 (10)
C6	0.0291 (15)	0.0226 (13)	0.0248 (13)	0.0045 (12)	0.0021 (12)	0.0015 (11)
C7	0.0252 (15)	0.0266 (14)	0.0265 (14)	0.0027 (12)	0.0022 (12)	−0.0005 (11)

### Geometric parameters (Å, º)

**Table:**

S1—C2	1.752 (2)	C3—H3A	0.9900
S1—C4	1.839 (3)	C3—H3B	0.9900
S2—C2	1.739 (3)	C4—C5	1.514 (4)
S2—C3	1.812 (3)	C4—H4	1.0000
O1—N1	1.243 (3)	C5—H5A	0.9900
O2—N1	1.241 (3)	C5—H5B	0.9900
N1—C1	1.414 (3)	C6—H6A	0.9800
N2—C5	1.466 (3)	C6—H6B	0.9800
N2—C7	1.466 (3)	C6—H6C	0.9800
N2—C6	1.467 (3)	C7—H7A	0.9800
C1—C2	1.358 (3)	C7—H7B	0.9800
C1—H1	0.9500	C7—H7C	0.9800
C3—C4	1.516 (4)		
			
C2—S1—C4	94.61 (12)	C3—C4—S1	105.80 (18)
C2—S2—C3	95.60 (12)	C5—C4—H4	109.5
O2—N1—O1	123.1 (2)	C3—C4—H4	109.5
O2—N1—C1	119.5 (2)	S1—C4—H4	109.5
O1—N1—C1	117.4 (2)	N2—C5—C4	111.3 (2)
C5—N2—C7	110.0 (2)	N2—C5—H5A	109.4
C5—N2—C6	111.9 (2)	C4—C5—H5A	109.4
C7—N2—C6	109.6 (2)	N2—C5—H5B	109.4
C2—C1—N1	121.8 (2)	C4—C5—H5B	109.4
C2—C1—H1	119.1	H5A—C5—H5B	108.0
N1—C1—H1	119.1	N2—C6—H6A	109.5
C1—C2—S2	126.56 (19)	N2—C6—H6B	109.5
C1—C2—S1	118.00 (19)	H6A—C6—H6B	109.5
S2—C2—S1	115.43 (14)	N2—C6—H6C	109.5
C4—C3—S2	108.41 (18)	H6A—C6—H6C	109.5
C4—C3—H3A	110.0	H6B—C6—H6C	109.5
S2—C3—H3A	110.0	N2—C7—H7A	109.5
C4—C3—H3B	110.0	N2—C7—H7B	109.5
S2—C3—H3B	110.0	H7A—C7—H7B	109.5
H3A—C3—H3B	108.4	N2—C7—H7C	109.5
C5—C4—C3	114.2 (2)	H7A—C7—H7C	109.5
C5—C4—S1	108.15 (18)	H7B—C7—H7C	109.5
			
O2—N1—C1—C2	−5.7 (4)	S2—C3—C4—C5	166.65 (18)
O1—N1—C1—C2	173.5 (2)	S2—C3—C4—S1	47.8 (2)
N1—C1—C2—S2	1.8 (4)	C2—S1—C4—C5	−161.36 (17)
N1—C1—C2—S1	−178.79 (18)	C2—S1—C4—C3	−38.61 (19)
C3—S2—C2—C1	−174.4 (2)	C7—N2—C5—C4	159.4 (2)
C3—S2—C2—S1	6.22 (17)	C6—N2—C5—C4	−78.6 (3)
C4—S1—C2—C1	−162.7 (2)	C3—C4—C5—N2	−173.9 (2)
C4—S1—C2—S2	16.82 (16)	S1—C4—C5—N2	−56.4 (2)
C2—S2—C3—C4	−33.5 (2)		

### Hydrogen-bond geometry (Å, º)

**Table:**

*D*—H···*A*	*D*—H	H···*A*	*D*···*A*	*D*—H···*A*
C1—H1···N2^i^	0.95	2.44	3.354 (4)	161
C4—H4···O1^ii^	1.00	2.44	3.272 (4)	140

Symmetry codes: (i) *x*−1/2, −*y*+3/2, −*z*; (ii) −*x*−1/2, −*y*+2, *z*+1/2.
